# Supplementary material for: Missing self triggers NK cell-mediated chronic vascular rejection of solid organ transplants
Source: Nat Commun. 2019 Nov 25;10:5350. doi: 10.1038/s41467-019-13113-5 (PMC6877588; doi:10.1038/s41467-019-13113-5)
Supplement: Supplementary file 1 — Supplementary Information [file 41467_2019_13113_MOESM1_ESM.pdf]

Supplementary information of manuscript entitled: “Missing self triggers NK cell-mediated chronic vascular rejection of solid organ transplants”

Koenig et al.

Supplementary Figures

Fig S1. Detection of non-HLA antibodies

Fig S2. Computer-assisted analysis of allograft inflammation (CAGI)

Fig S3. Definition of inhibitory KIR repertoire

Fig S4. Gating strategies used for flow cytometry analyses

Fig S5. Allogeneic endothelial cells trigger missing self-induced activation of NK cells *in vitro*

Fig S6. Missing self-induced activation of NK cells has a deleterious impact on endothelial cells

Fig S7. Missing self triggers NK cell-mediated rejection *in vivo*

Fig S8. Gating strategy used for the analysis of activated NK cells by imaging flow cytometry

Supplementary Tables

Table S1. Clinical characteristics of renal transplant patients

Table S2. HLA and KIR genotypes of donors & recipients

Supplementary figures

Supplementary Figure 1: Detection of non-HLA antibodies

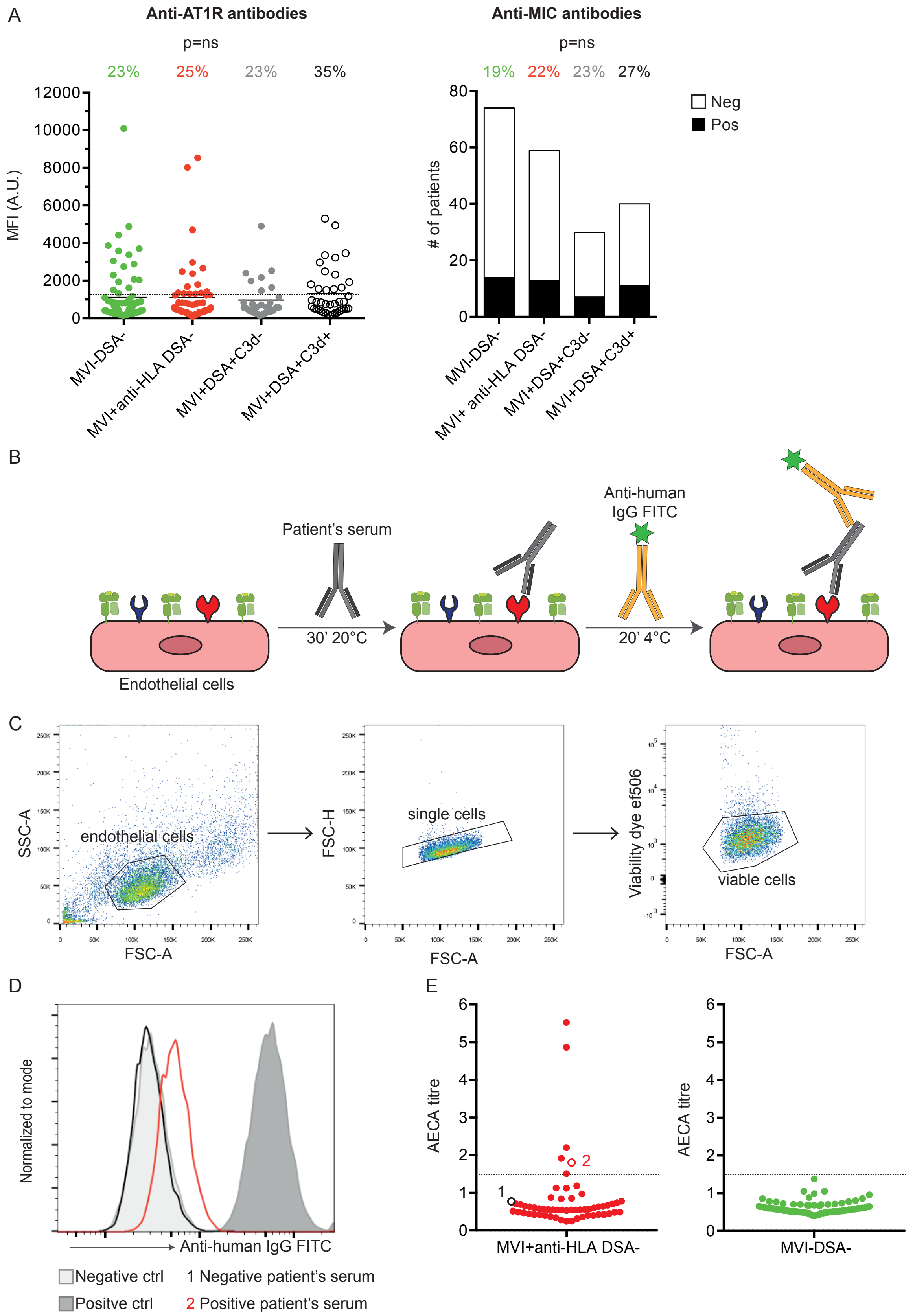

A. Histograms showing individual data for the screening of anti-AT1R (left) and anti-MIC (right) antibodies in the sera of MVI-DSA- (n=74), MVI+ anti-HLA DSA- (n=59), MVI+DSA+C3d- (n=30) and MVI+DSA+C3d+ (n=40) patients.

B. Schematic representation of the flow cross match technique used to detect anti-endothelial antibodies (AECA) in the circulation of renal transplant patients.

C. Gating strategy used to assess the presence of non-HLA antibodies by endothelial flow cross match.

D. Overlay of representative flow cytometry profiles of endothelial flow cross matches. Shaded profiles are respectively for endothelial cells incubated in AB serum (negative control, light grey) or in serum of patients with anti-HLA antibodies (positive control, dark grey). Open profiles are representative of a negative (patient #1, black) and a positive (patient #2, red) test, respectively.

E. Histograms showing the individual endothelial flow cross match result from patients of MVI+ anti-HLA DSA- (left, n=59) and MVI-DSA- (right, n=72) group. AECA titre is the ratio of mean fluorescence intensities of endothelial cells incubated in patient's serum and AB serum (negative control). Grey dashed line indicates the threshold of positivity of the assay (AECA titre > 1.5).

Supplementary Figure 2: Computer-assisted analysis of allograft inflammation (CAGI)

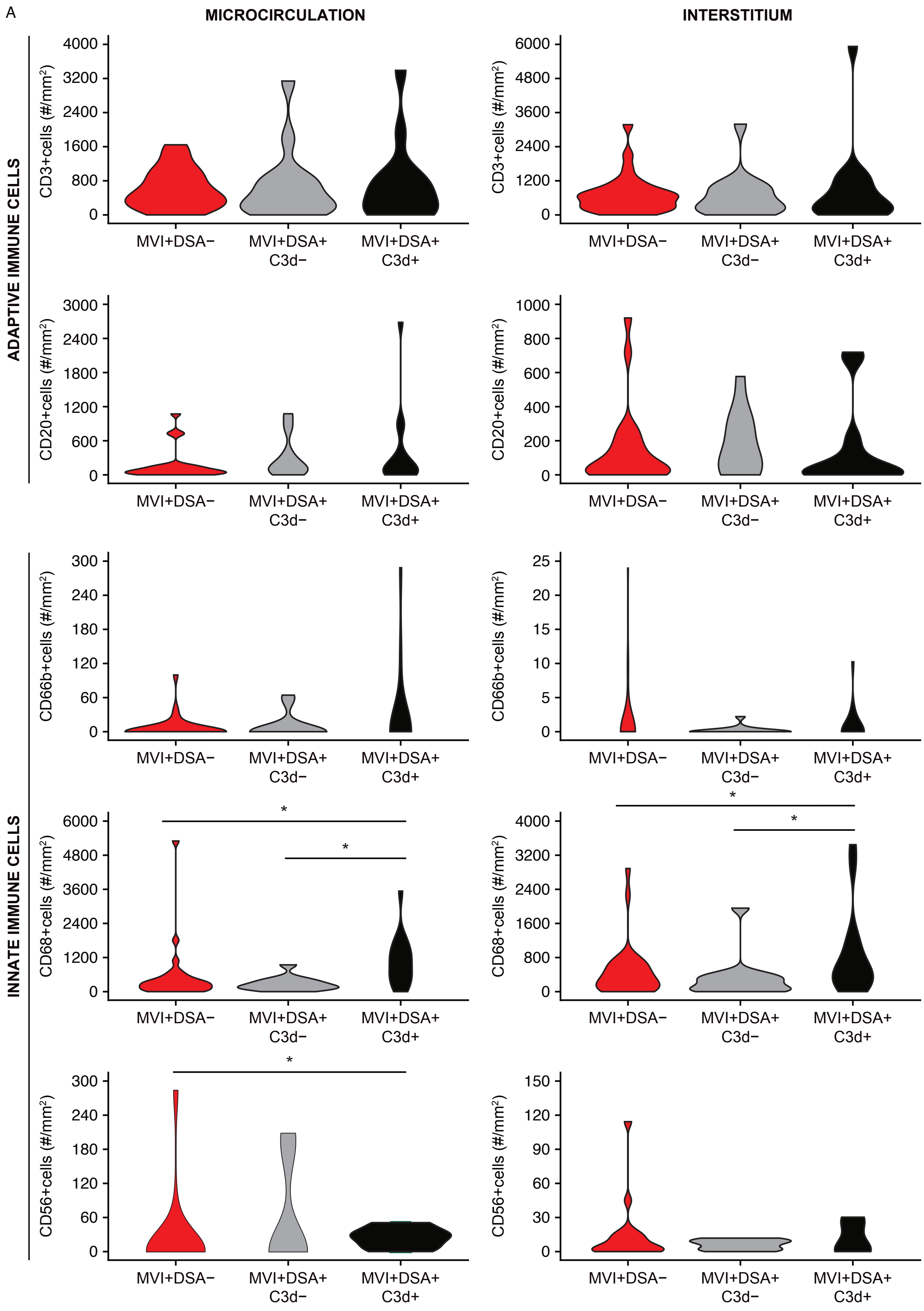

A. Quantification of adaptive effectors (B cells [CD20+], and T cells [CD3+]) and innate effectors (macrophages [CD68+], granulocytes [CD66b+], and NK cells [CD56+]) in the microcirculation (glomeruli and peritubular capillaries) and the tubulo-interstitial compartment of renal allograft of patients with available biopsy material from MVI+DSA+C3d+ (n=17), MVI+DSA+C3d- (n=14), and MVI+DSA- (n=32) groups using the Computer-assisted Analysis of Graft Inflammation (CAGI) method. *: $p<0.05$; One-way Anova.

Supplementary Figure 3: Definition of inhibitory KIR repertoire

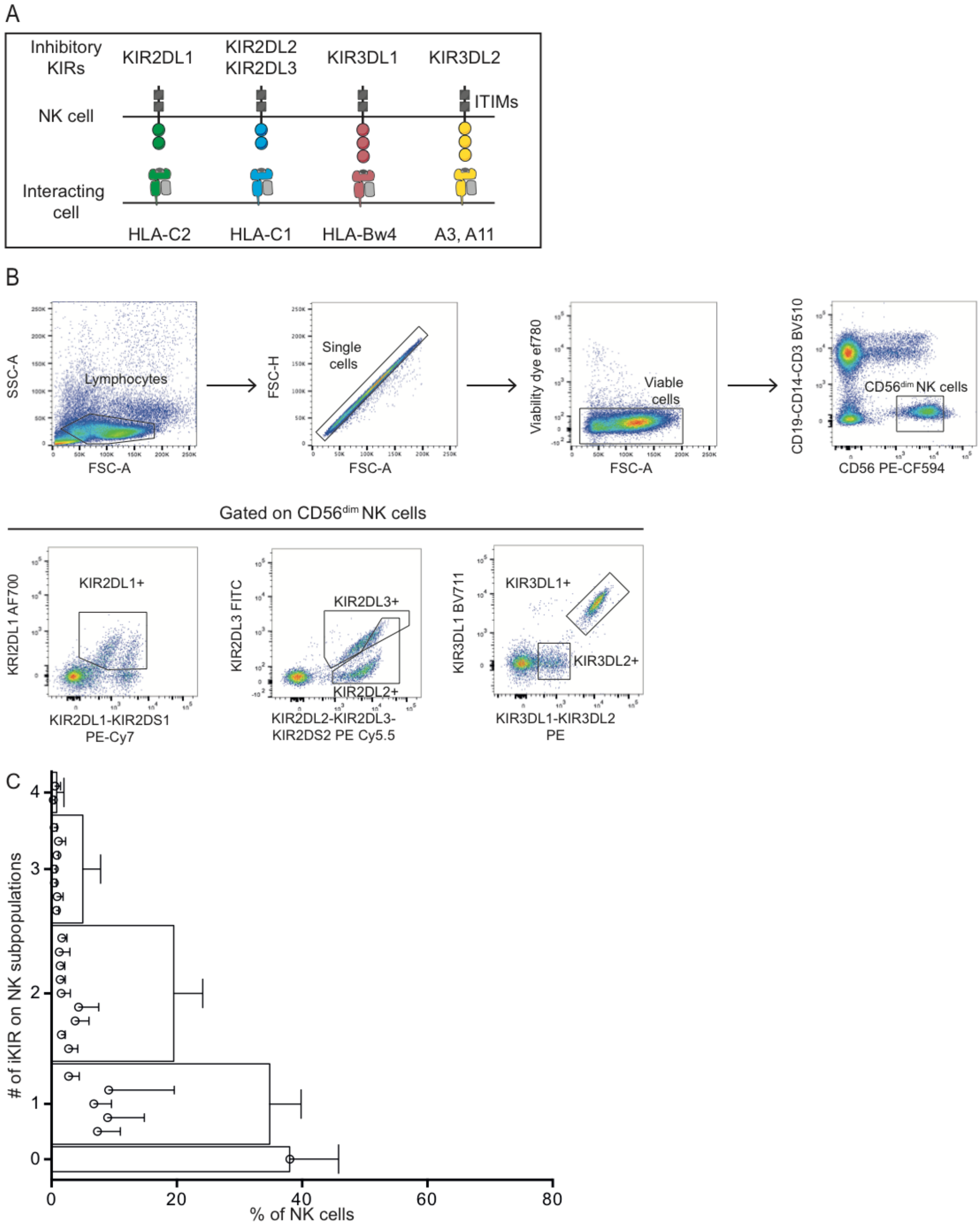

A. Schematic representation of the interactions between inhibitory KIR receptors on human NK cells and their ligands. KIR2DL1 and KIR2DL2/3 recognise distinct HLA-C allotypes, called C2 or C1 based on polymorphisms at positions 77 and 80 in the α1-domain of the HLA heavy chain. KIR3DL1 ligands are HLA A and B molecules that share the Bw4 epitope, and KIR3DL2 binds HLA-A3 and HLA-A11.

B-C Flow cytometry was used to analyse the expression of the 5 inhibitory KIRs (2DL1, 2DL2, 2DL3, 3DL1, 3DL2) on circulating NK cells of 6 healthy volunteers with identical genotypes. **B.** A combination of 6 fluorescent mAb specific for respectively i) KIR2DL1 and KIR2DS5, ii) KIR2DL1, iii) KIR2DL2 and KIR2DL3 and KIRD2DS2, iv) KIR2DL3, v) KIR3DL1 and KIR3DL2, and vi) KIR3DL1 were used. The gating strategy used to identify NK cell populations that express a single inhibitory KIR is shown. **C.** According to the combination of inhibitory KIRs expressed on the cell surface, 23 subsets of NK cells could be defined. The histogram shows the relative proportion of each NK cell subset: i.e. inhibitory KIR repertoire. The open circle indicates the mean of each NK cell subset. Bars indicate the mean of each category, which were defined according to the number of inhibitory KIR expressed on the NK cell surface (0 to 5). Standard deviation is indicated for each mean value. Source data are provided as a Source Data file.

Supplementary Figure 4: Gating strategies used for flow cytometry analyses

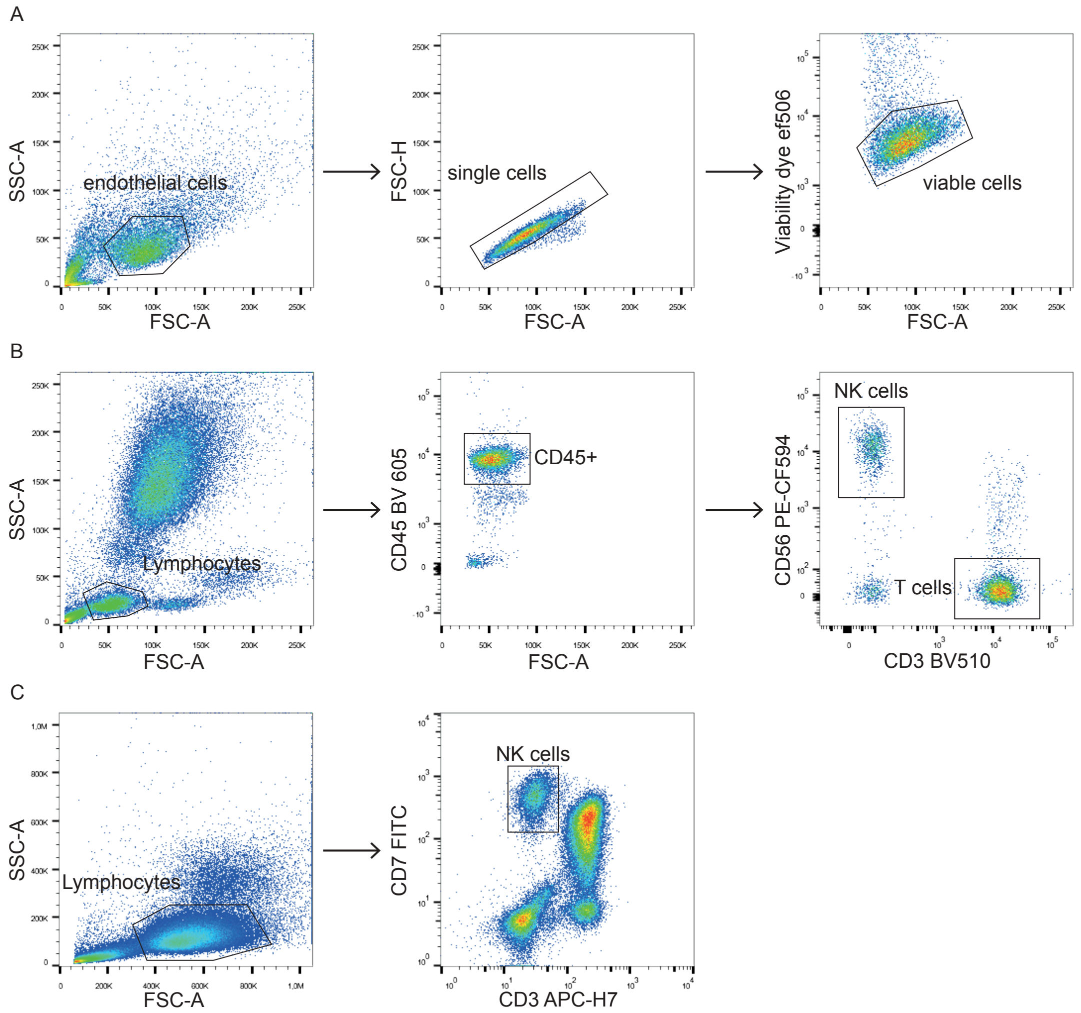

The gating strategies used to **A.** analyse the expression of HLA class I on endothelial cells, **B**. to count NK cells and T cells in the blood of healthy volunteers, and **C.** to measure the expression of phosphorylated form of S6 Ribosomal Protein (S6RP) in NK cells by flow cytometry are shown.

Supplementary Figure 5: Allogeneic endothelial cells trigger missing self-induced activation of NK cells *in vitro*

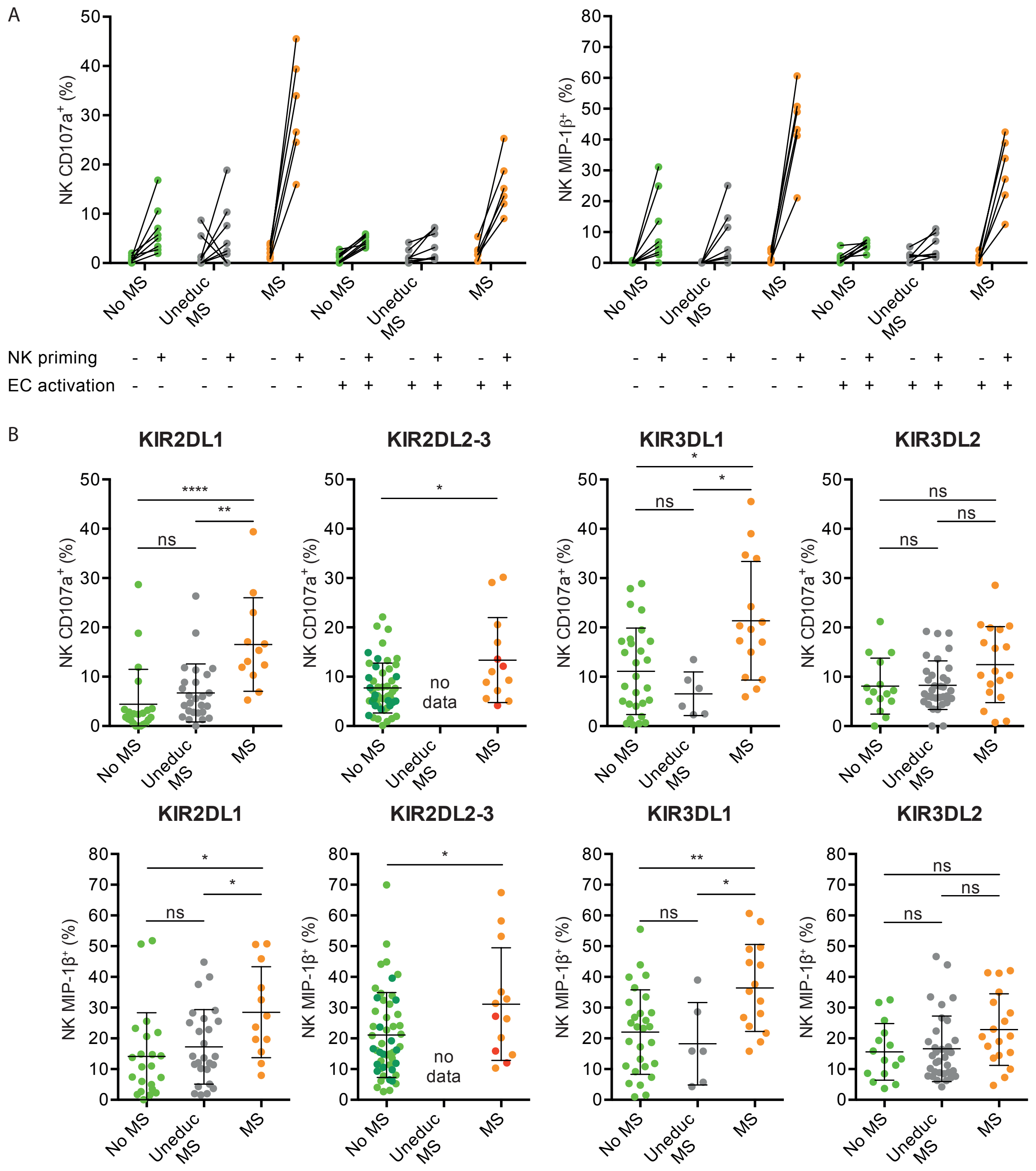

Primary allogeneic human endothelial cells were co-cultured with purified NK cells from 30 healthy volunteers. After 4 hours of culture, the activation status of the NK cells was assessed at the single cell level by flow cytometry focusing on the 5 NK cell populations that expressed a single inhibitory KIR. Three distinct situations were

defined: i) endothelial cells expressed the ligand for the inhibitory KIR (No MS), ii) neither endothelial cells nor NK cell donor expressed the ligand for the inhibitory KIR (Uneduc MS), or iii) endothelial cells did not expressed the ligand of inhibitory KIR (missing self, MS).

A. Experiments were performed with purified NK cells unprimed or primed with low dose IL-2 in presence of endothelial cells activated or not with TNF α. Left: Expression of CD107a (LAMP-1) on NK cells surface. Right: Intracellular staining for MIP-1β in NK cells.

B. Experiments were performed after priming of purified NK cells with low dose IL-2 in the presence of non-activated endothelial cells. Each graph shows the ability of a given missing self situation to activate NK cells.

KIR2DL2 (red or dark green) and KR2DL3 (orange or light green) were plotted together because they bind to the same ligand on endothelial cells. Upper row: Expression of CD107a (LAMP-1) on NK cells surface. Lower row: Intracellular staining for MIP-1β in NK cells.

ns: $p \geq 0.05$; *: $p < 0.05$; **: $p < 0.01$; ****: $p < 0.0001$; One-way Anova.

Supplementary Figure 6: Missing self-induced activation of NK cells has a deleterious impact on endothelial cells

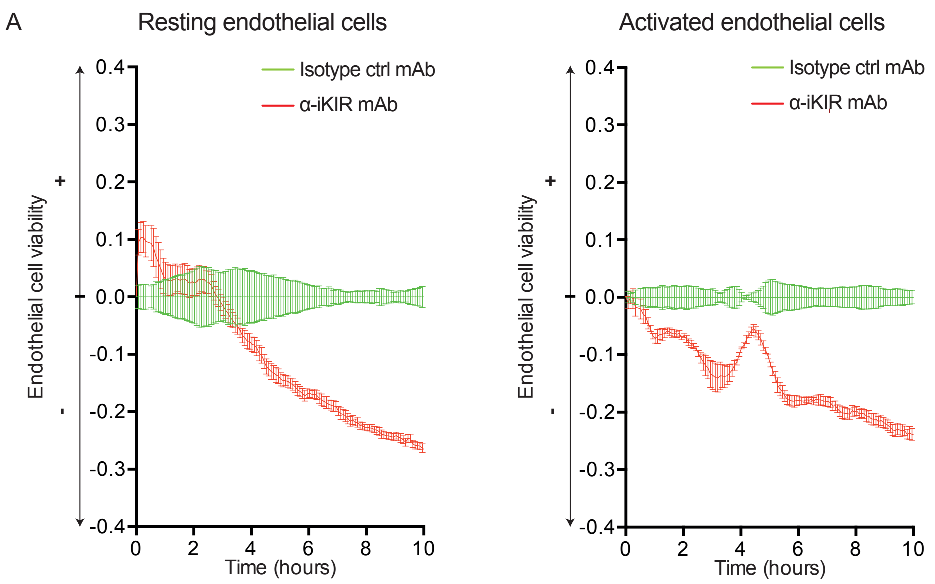

A. Purified NK cells from a donor without missing-self were co-cultured with the same primary allogeneic human endothelial cells activated or not with TNF α, in presence of a blocking anti-inhibitory KIR mAb (experimental co-culture) or an isotype control mAb (control coculture). The viability of endothelial cells was assessed by real-time impedance measurement. Data of the experimental co-culture was normalized over control. Individual impedance profiles (mean ± standard error) are shown.

Supplementary Figure 7: Missing self triggers NK cell-mediated rejection *in vivo*

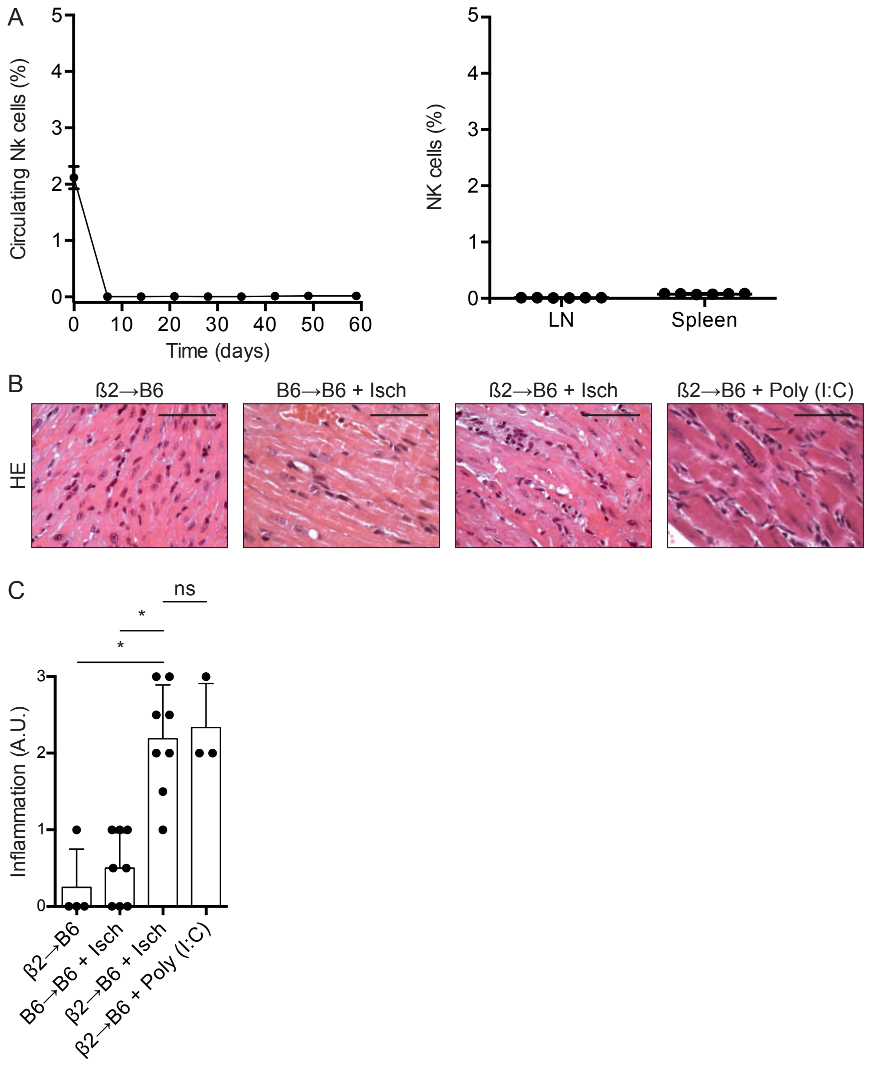

A. The efficiency of the NK cell depletion was assessed in recipient mice by flow cytometry. Mice transplanted with a β2 microglobulin KO heart allograft were depleted for NK cells by IP injection of 100 µg anti-NK1.1 mAb twice a week from day -7 to day 60 post transplantation. Left: the proportion of Nkp46+ cells was assessed in the circulation of 5 recipient mice before (0) depletion and every 7 days post-transplantation. Mean ± standard deviation. Right: at day 60 post-transplantation (the time when heart graft was harvested for pathological analyses), NK cell depletion

was assessed in secondary lymphoid organs (LN: lymph nodes, and spleen) of recipient mice. Individual data of 6 mice are shown.

B-C. Wild type C57BL/6 mice were transplanted with either a C57BL/6 or a β2 microglobulin KO heart. In some cases the heart was subjected to 3 hours of cold ischemia before transplantation (+ Isch). Some recipients received 100 µg of polyinosinic-polycytidylic acid IP 4 days post-transplantation (+ Poly(I:C)). Heart grafts were harvested 60 days after transplantation for histological analysis. **D.** Representative findings of H&E stain are shown for the 4 experimental groups. Scale bars: 100µm. **E.** A trained pathologist graded the intensity of the microvascular inflammation (MVI) on a semi-quantitative scale (score 0–3). Mean ± standard deviation.

ns: $p \geq 0.05$; *: $p < 0.05$; One-way Anova. Source data are provided as a Source Data file.

Supplementary Figure 8: Gating strategy used for the analysis of activated NK cells by imaging flow cytometry

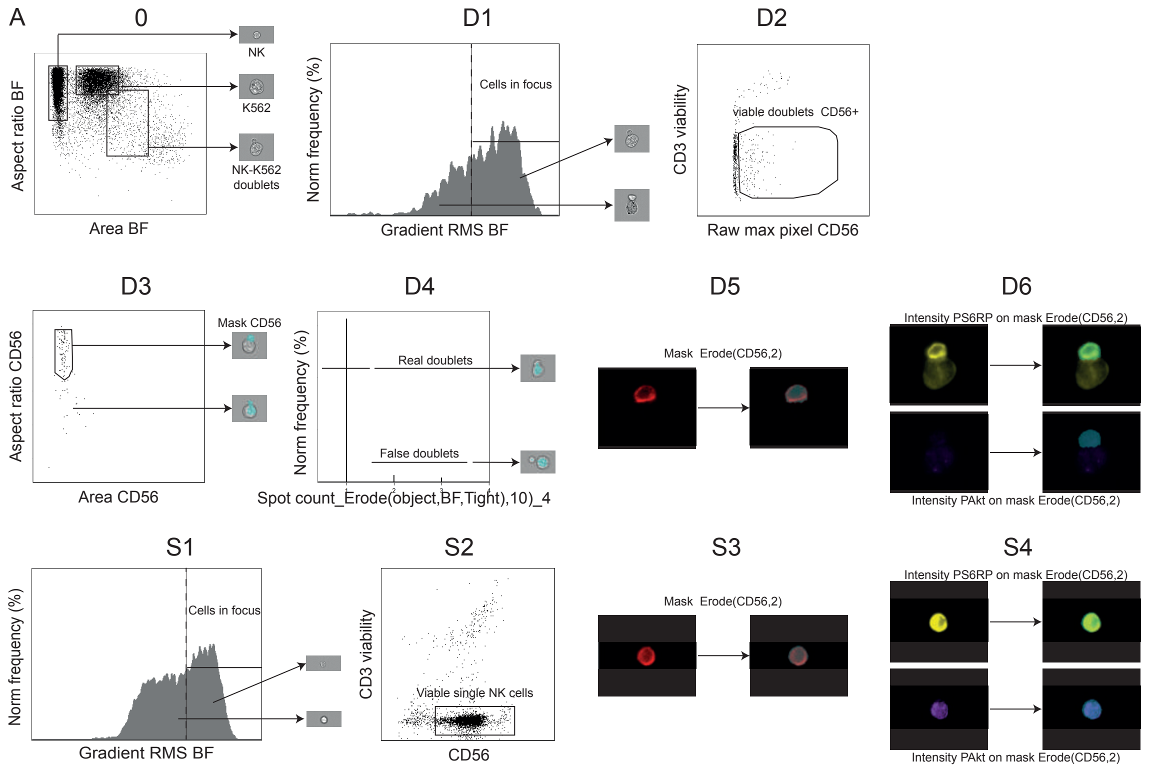

Purified NK cells from a healthy donor were co-cultured with HLA-deficient K562 cells. Imaging flow cytometer was used to detect the phosphorylated form of S6 Ribosomal Protein (S6RP, downstream mTORC1) and protein kinase B (Akt, downstream mTORC2) in isolated NK cells and NK cells forming doublets with K562 target cells.

A. The following populations were gated: (0) single NK cells (S) and doublets formed by an NK cell and a K562 cell (D) by BF Area (x-axis) and Aspect Ratio (y axis). Selection of doublets: (D1) elimination of out of focus cells. (D2 to D4) Elimination of CD3+ T cells and dead cells and identification of doublets of NK (CD3-CD56+) and K562 cells. (D5) CD56 mask. (D6) Upper row: representative example of p-S6RP signal in NK/K562 doublets (left) and overlay between CD56 mask (area where the

signal intensity was measured) and p-S6RP signal. Lower row: representative example of p-Akt signal in NK/K562 doublets (left) and overlay between CD56 mask and p-Akt signal.

Selection of single NK cells: (S1) elimination of out of focus cells. (S2) Elimination of CD3+ T cells and dead cells and identification of single NK cells (CD3-CD56+). (S3) CD56 mask. (S4) Upper row: representative example of p-S6RP signal in single NK cells (left) and overlay between CD56 mask and p-S6RP signal. Lower row: representative example of p-Akt signal in in single NK cells (left) and overlay between CD56 mask and p-Akt signal.

Supplementary Tables

Supplementary Table 1: Clinical characteristics of renal transplant patients

Variable	MVI+DSA+C3d+ (n=40)	MVI+DSA+C3d- (n=30)	MVI+DSA- (n=43)	MVI-DSA- (n=55)	*P* value 2 vs 3	*P* value 3 vs 4
**Characteristics at the time of transplantation**						
Recipient						
Age (yr)	36.7 ± 12.4	43.7 ± 15.5	42.3 ± 13.7	45.4 ± 14.0	0.7	0.3
Men, *n* (%)	26 (65.0)	17 (56.7)	30 (69.8)	42 (76.4)	0.3	0.5
Retransplantation, *n* (%)	14 (35.0)	10 (33.3)	10 (23.3)	5 (9.1)	0.4	0.1
Time since dialysis (mo)	50.2 ± 65.1	61.0 ± 64.9	25.3 ± 29.1	29.4 ± 34.6	0.002	0.5
Blood group, n (%)						
Type A	24 (60.0)	15 (50.0)	23 (53.5)	25 (45.5)	0.2	0.02
Type B	2 (5.0)	4 (13.3)	6 (14.0)	8 (14.5)		
Type O	13 (32.5)	11 (36.7)	9 (20.9)	22 (40.0)		
Type AB	1 (2.5)	0 (0.0)	5 (11.6)	0 (0.0)		
Donor						
Deceased, *n* (%)	37 (92.5)	28 (93.3)	40 (93.0)	49 (89.1)	1.0	0.7
Age (yr)	38.1 ± 17.9	40.2 ± 15.9	43.3 ± 14.5	45.5 ± 16.2	0.4	0.5
**Transplantation**						
Cold ischemia time (min)	902 ± 396	1024 ± 288	1013 ± 344	876 ± 307	0.9	0.03
No. of HLA A/B/DR mismatch	3.6 ± 1.5	4.0 ± 1.3	4.0 ± 1.3	3.9 ± 1.4	1.0	0.8
Combined transplantation, *n* (%)[a]	5 (12.5)	3 (10.0)	6 (14.0)	15 (27.3)	0.7	0.1
Delayed graft function, *n* (%)	10 (25.0)	4 (13.3)	7 (16.7)	9 (16.4)	0.8	1.0
**Immunosuppression**						
**Induction therapy**						
Antithymocyte globulins	30 (75.0)	23 (76.7)	28 (65.1)	47 (85.5)	0.4	0.04
Anti-IL2 receptor	6 (15.0)	4 (13.3)	14 (32.6)	14 (25.5)	0.06	0.5
**Maintenance therapy**						
Cyclosporine	23 (57.5)	18 (60.0)	17 (39.5)	16 (29.1)	0.1	0.3
Tacrolimus	15 (37.5)	11 (36.7)	25 (58.1)	40 (72.7)	0.1	0.1
Azathioprine	4 (10.0)	1 (3.0)	1 (2.3)	0 (0.0)	1.0	0.4
Mycophenolate mofetif	33 (82.5)	26 (90.0)	41 (95.3)	55 (100)	0.4	0.2
Steroids	38 (95.0)	29 (96.7)	42 (97.7)	54 (98.2)	1.0	1.0
**Characteristics at the time of biopsy**						
Clinicobiologic characteristics						
Time post-transplantation (mo)	55 ± 59.9	38.9 ± 41.4	25.1 ± 39.7	8.5 ± 5.9	0.2	0.003
Proteinuria (g/d)	2.3 ± 5.8	0.7 ± 1.0	0.7 ± 1.5	0.1 ± 0.2	0.9	0.01
Creatininemia (µmol/L)	362 ± 382	207 ± 138	186 ± 86	137 ± 57	0.4	0.001
eGFR[b] (ml/min per 1.73 m^2)	29.5 ± 20.9	38.4 ± 18.6	43.4 ± 21.1	56.6 ± 20.6	0.4	0.003
Histologic characteristics (Banff scores[c])						
Microvascular inflammation[d]	3.5 ± 1.2	3.4 ± 1.0	3.0 ± 1.3	0.2 ± 0.4	0.2	< 0.0001
Transplant glomerulopathy	1.1 ± 1.2	1.0 ± 1.2	1.0 ± 1.3	0.02 ± 0.1	0.9	< 0.0001
Interstitial inflammation and tubulitis	2.9 ± 2.1	2.3 ± 1.8	2.3 ± 1.7	1.5 ± 1.9	0.9	0.03
Interstitial fibrosis and tubular atrophy	1.7 ± 0.8	1.5 ± 0.7	1.7 ± 1.0	1.4 ± 0.7	0.4	0.06
Arteriosclerosis	0.9 ± 1.0	1.1 ± 1.1	1.2 ± 1.1	0.7 ± 0.8	0.9	0.02
Endarteritis (vasculitis)	0.25 ± 0.5	0.3 ± 0.5	0.3 ± 0.7	0	0.8	n/a
C4d deposition	1.75 ± 0.9	1.3 ± 1.1	0.3 ± 0.7	0	< 0.0001	n/a

Abbreviations: DSA: Donor specific antibodies, MVI: microvascular inflammation
a Simultaneous pancreas and kidney transplantations.
b Calculated with the Modification of Diet in Renal Disease formula.
c Banff scores (0: no significant lesion, 1: mild, 2: moderate, 3: severe).
d Sum of the Banff scores for glomerulitis and capillaritis.
Source data are provided as a Source Data file.

Supplementary Table 2: HLA and KIR genotypes of donors & recipients

	MVI-DSA- (n=55)	MVI+DSA- (n=43)	*P* value
**RECIPIENTS**			
**KIR genes**			
**Inhibitory KIRs, *n* (%)**			
KIR2DL1	53 (96.4)	41 (95.3)	1.0
KIR2DL2	31 (56.4)	21 (46.5)	0.4
KIR2DL3	49 (89.1)	40 (93.0)	0.7
KIR2DL5	32 (58.2)	19 (44.2)	0.2
KIR3DL1	52 (94.5)	43 (100.0)	0.3
KIR3DL2	55 (100.0)	43 (100.0)	1.0
KIR3DL3	55 (100.0)	43 (100.0)	1.0
**Activating KIRs, *n* (%)**			
KIR2DS1	17 (30.9)	12 (27.9)	0.8
KIR2DS2	31 (56.4)	20 (46.5)	0.4
KIR2DS3	21 (38.0)	7 (16.3)	0.02
KIR2DS4	52 (94.5)	43 (100.0)	0.3
KIR2DS5	18 (32.7)	13 (30.2)	0.8
KIR3DS1	15 (27.3)	12 (27.9)	1.0
**Unknown, *n* (%)**			
KIR2DL4	55 (100.0)	43 (100.0)	1.0
**Numbers of KIRs**			
**Inhibitory KIRs, *n* (%)**			
5	16 (29.1)	17 (39.5)	0.4
6	21 (38.2)	17 (39.5)	
7	17 (30.9)	9 (21.0)	
**Activating KIRs, *n* (%)**			
1-2	22 (40.0)	24 (55.8)	0.4
3-4	23 (41.8)	16 (37.2)	
5-6	8 (14.5)	3 (7.0)	
**Haplotypes, *n* (%)**			
A/A	14 (25.5)	14 (32.6)	0.2
B/B	3 (5.4)	0 (0.0)	
A/B	38 (69.1)	29 (67.4)	
**Inhibitory KIR ligands, n (%)**			
C1/C1	21 (38.2)	16 (37.2)	1.0
C1/C2	25 (45.5)	19 (44.2)	
C2/C2	9 (16.3)	8 (18.6)	
Bw4	41 (74.5)	35 (81.4)	0.5
A3, A11	17 (30.9)	9 (20.9)	0.5
**Educating inhibitory KIRs, *n* (%)**			
KIR2DL1+/C2+	32 (58.2)	26 (60.4)	0.8
KIR2DL2+/C1+	24 (43.6)	16 (37.2)	0.5
KIR2DL3+/C1+	42 (76.4)	32 (74.4)	1.0
KIR3DL1+/Bw4+	38 (69.1)	35 (81.4)	0.2
KIR3DL2+/A 3, 11+	15 (27.3)	10 (23.3)	0.8
**DONORS**			
**Inhibitory KIR ligands, n (%)**			
C1/C1	18 (32.7)	17 (39.5)	0.2
C1/C2	32 (58.2)	18 (41.9)	
C2/C2	5 (9.1)	8 (18.6)	
Bw4	45 (81.8)	27 (62.8)	0.04
A3, A11	26 (47.3)	11 (25.6)	0.04

Abbreviations: DSA: Donor specific antibodies, MVI: microvascular inflammation
