# Supplementary material for: Missing self triggers NK cell-mediated chronic vascular rejection of solid organ transplants
Source: Nat Commun. 2019 Nov 25;10:5350. doi: 10.1038/s41467-019-13113-5 (PMC6877588; doi:10.1038/s41467-019-13113-5)
Supplement: Supplementary file 3 — Reporting Summary [file 41467_2019_13113_MOESM3_ESM.pdf]

nature research

Corresponding author(s): Olivier Thaunat

Reporting Summary

Nature Research wishes to improve the reproducibility of the work that we publish. This form provides structure for consistency and transparency in reporting. For further information on Nature Research policies, see Authors & Referees and the Editorial Policy Checklist.

Statistical parameters

When statistical analyses are reported, confirm that the following items are present in the relevant location (e.g. figure legend, table legend, main text, or Methods section).

n/a	Confirmed	
☐	☒	The exact sample size (n) for each experimental group/condition, given as a discrete number and unit of measurement
☐	☒	An indication of whether measurements were taken from distinct samples or whether the same sample was measured repeatedly
☐	☒	The statistical test(s) used AND whether they are one- or two-sided<br>*Only common tests should be described solely by name; describe more complex techniques in the Methods section.*
☐	☒	A description of all covariates tested
☐	☒	A description of any assumptions or corrections, such as tests of normality and adjustment for multiple comparisons
☐	☒	A full description of the statistics including central tendency (e.g. means) or other basic estimates (e.g. regression coefficient) AND variation (e.g. standard deviation) or associated estimates of uncertainty (e.g. confidence intervals)
☐	☒	For null hypothesis testing, the test statistic (e.g. F, t, r) with confidence intervals, effect sizes, degrees of freedom and P value noted<br>*Give P values as exact values whenever suitable.*
☒	☐	For Bayesian analysis, information on the choice of priors and Markov chain Monte Carlo settings
☒	☐	For hierarchical and complex designs, identification of the appropriate level for tests and full reporting of outcomes
☒	☐	Estimates of effect sizes (e.g. Cohen's d, Pearson's r), indicating how they were calculated
☐	☒	Clearly defined error bars<br>*State explicitly what error bars represent (e.g. SD, SE, CI)*

Our web collection on statistics for biologists may be useful.

Software and code

Policy information about availability of computer code

Data collection	n/a
Data analysis	n/a

For manuscripts utilizing custom algorithms or software that are central to the research but not yet described in published literature, software must be made available to editors/reviewers upon request. We strongly encourage code deposition in a community repository (e.g. GitHub). See the Nature Research guidelines for submitting code & software for further information.

Data

Policy information about availability of data

All manuscripts must include a data availability statement. This statement should provide the following information, where applicable:

- Accession codes, unique identifiers, or web links for publicly available datasets
- A list of figures that have associated raw data
- A description of any restrictions on data availability

The source data underlying Figure 2c, Figure 3c & 3d, Figure 6b & 6c, Figure 7b, Supplementary figure 3c, Supplementary figure 6a, Supplementary table 1 are provided as a source data file

The authors declare that all data supporting the findings of this study are available within the article and its supplementary information, or are available on request from the corresponding author.

Field-specific reporting

Please select the best fit for your research. If you are not sure, read the appropriate sections before making your selection.

☒ Life sciences ☐ Behavioural & social sciences ☐ Ecological, evolutionary & environmental sciences

For a reference copy of the document with all sections, see nature.com/authors/policies/ReportingSummary-flat.pdf

Life sciences study design

All studies must disclose on these points even when the disclosure is negative.

Sample size	For the clinical cohort , 198 patients were eligible (40 in the group MVI+DSA+C3d+; 30 in the group MVI+DSA+C3d-; 53 in the group MVI+DSA- and 75 in the group MVI-DSA-) but only 168 were finally included.
Data exclusions	30 patients were excluded (10 in the group MVI+DSA- and 20 in the group MVI-DSA-) because DNA samples were not available for the donor and/or the recipient. Without this information, the analysis of missing self was impossible.
Replication	All in vitro and in vivo experiments have been replicated at least twice.
Randomization	n/a
Blinding	n/a

Reporting for specific materials, systems and methods

Materials & experimental systems

n/a	Involved in the study	
☒	☐	Unique biological materials
☐	☒	Antibodies
☐	☒	Eukaryotic cell lines
☒	☐	Palaeontology
☐	☒	Animals and other organisms
☐	☒	Human research participants

Methods

n/a	Involved in the study	
☒	☐	ChIP-seq
☐	☒	Flow cytometry
☒	☐	MRI-based neuroimaging

Antibodies

Antibodies used	Antibodies directed against the following human epitopes were used in flow cytometry : CD45 (clone 30-F11, 1/400, BioLegend, London, UK), CD3 (clone SK7, 1/10, BD biosciences), CD56 (clone NCAM16.2, 1/10, BD biosciences), CD7 (clone 8H8.1, 1/50, Beckman Coulter), CD19 (clone HIB19, 1/10, BD biosciences), CD14 (clone M5E2, 1/10, BD biosciences), KIR3DL1 (clone DX9, 1/25, BD biosciences), KIR2DL1/S5 (clone 143211, 1/10, R&Dsystems), KIR2DL3 (clone 180701,1/10, R&Dsystems) KIR2DL1/S1 (clone EB6B, 1/25, Beckman Coulter), KIR2DL2-3/S2 (clone GL183, 1/25, Beckman Coulter), KIR3DL1-2 (clone REA168, 1/10, Miltenyi Biotec), Phospho-S6 Ribosomal Protein Ser 235/236 (clone D57.2.2E, 1/50, Cell Signaling Technology), PAkt S473 (clone M89-61, 1/40, BD biosciences), CD107a-FITC (clone H4A3, 5 µl, ThermoFisher Scientific), MIP1ß-V450 (clone D21-1351, 1/40, BD biosciences),<br>Antibodies directed against the following human epitopes were used in immunochemistry : CD34 (clone QBEnd10, 1/200, Dako, Les Ulis, France) , CD3 (clone SK7, 1/150, Becton Dickinson, Le Pont de Claix, France), CD20 (clone L26, 1/400, Dako), CD66b (clone G10F5, 1/300, Becton Dickinson), CD68 (clone PGM1, 1/100, Dako) and CD56 (clone CD564, 1/10, produced by Novocastra and distributed by Leica Microsystemes SAS, Nanterre, France)<br>Antibodies against the following mouse epitopes were used in immunochemistry: CD31 (clone SZ31; 1/50; Dianova), CD45 (clone 30-F11; 1/40, BD biosciences), anti-Nkp46 (kind gift from Innate Pharma, Marseille, France),<br>Antibodies against the following mouse epitopes were injected in vivo to mice: NK1.1 (clone PB136, 200 µg by injection, BioXcell)
Validation	All the antibodies were commercial ones validated for the applications in which they were used.

Eukaryotic cell lines

Policy information about cell lines

Cell line source(s)	Human erythroleukemia cell line K562 were kindly gifted by I. Doxiadis, University of Leiden, Netherlands who obtained the cells at the American Type Culture Collection (Manassas, VA)<br>Primary human arterial endothelial cells were isolated from organ donors (agreement PFS08-017 from the Agence de la Biomédecine, https://www.agence-biomedecine.fr) and prospectively stored in the DIVAT biobank (N° of biocollection #02G55)
Authentication	For K562, the absence of expression of HLA class I was verify at each experiment.<br>For primary endothelial cell lines, the expression of endothelial cell markers VE-cadherin, CD31 was verify at each experiment
Mycoplasma contamination	tested negative
Commonly misidentified lines (See ICLAC register)	n/a

Animals and other organisms

Policy information about studies involving animals; ARRIVE guidelines recommended for reporting animal research

Laboratory animals	Wild type female C57BL/6 (H-2b) mice aged 8-15 weeks were used.<br>ß2 microglobulin KO female C57BL/6 (H-2b) mice aged 6-12 weeks were used.
Wild animals	The study did not involved wild animals
Field-collected samples	The study did not involved samples collected from the field

Human research participants

Policy information about studies involving human research participants

Population characteristics	All the population characteristics are described in the supplementary table 1
Recruitment	Participants were recruited retrospectively:<br>The computer database (DIAMIC) of the Lyon University Hospital pathology department was used to screen all kidney-allograft biopsies (2024 biopsies in 938 patients) performed between September 1st 2004 and September 1st 2012, for microvascular inflammation (MVI+). The biopsies of the 143 patients were systematically reviewed by the same trained pathologist (M. Rabeyrin), who graded the lesions according to Banff 2011 classification. Fourteen patients, whose biopsy analysis did not confirm the presence of MVI lesions (Banff g+ptc score<2) were excluded.<br>Clinical data of the 129 patients enrolled in the study was obtained from two independent national registries [Cristal: http://www.sipg.sante.fr/portail/, and Données Informatiques Validées en Transplantation (DIVAT); http://www.divat.fr/] and crosschecked.<br>Serum samples banked at the time of biopsy were screened for the presence of anti-HLA donor-specific antibodies (DSA), and, if positive, for the ability of these anti-HLA DSA to bind the complement fraction C3d. These centralized analyses were performed in a blinded fashion with single-antigen flow bead assays according to the manufacturer's instructions (Immucor, Norcross, GA, USA). If negative, all the serum samples collected during the follow-up of the patients were checked to confirm this negativity. To rule out the presence of non-HLA donor-specific antibodies, negative sera were tested in endothelial flow cross match assay as described in reference 30 (see Supplementary methods section for details).<br>The steps leading to the distribution of patients into the first 3 groups of patients (MVI+DSA+C3d+, n=40; MVI+DSA+C3d-, n=30; and MVI+DSA-, n=53) are summarized Figure 1A.<br>A control group, without MVI on graft biopsy, nor circulating DSA (MVI-DSA-, n=75), but matched for the main clinical characteristics of the MVI+DSA- patients, was established from the pool of 938 patients.

Methodology

Sample preparation

NK cell count
Two hundred microliters of blood were incubated with anti-CD45 (clone 30-F11, 1/400, BioLegend, London, UK), -CD3 (clone SK7, 1/10, BD biosciences, Le Pont de Claix, France) and -CD56 (clone NCAM16.2, 1/10, BD biosciences) antibodies. The samples were then incubated with a Lysing Solution (BD biosciences) to eliminate the red blood cells. Lymphocyte count was performed with ABX Pentra 60C+ (Horiba, Irvine, CA, USA).

KIR phenotyping
Single cell suspensions of human PBMCs were incubated with a fixable viability dye (ThermoFisher Scientific) for 20 minutes at 4° C. After washing, the cells were incubated first with anti-CD19 (clone HIB19, 1/10, BD biosciences), -CD14 (clone M5E2, 1/10, BD biosciences), -CD3 (clone SK7, 1/10, BD biosciences), -CD56 (clone NCAM16.2, 1/10, BD biosciences), -KIR3DL1 (clone DX9, 1/25, BD biosciences), -KIR2DL1/S5 (clone 143211, 1/10, R&Dsystems), and -KIR2DL3 (clone 180701,1/10, R&Dsystems) antibodies for 15 minutes at room temperature and then with anti-KIR2DL1/S1 (clone EB6B, 1/25, Beckman Coulter, Villepinte, France), -KIR2DL2-3/S2 (clone GL183, 1/25, Beckman Coulter), and -KIR3DL1-2 (clone REA168, 1/10, Miltenyi Biotec, Bergisch Gladbach, Germany) antibodies for an additional 15 minutes. The cells were then fixed with paraformaldehyde 2 % (ThermoFisher Scientific) and the sample was stored at 4°C until analysis.

NK cell activation in vitro
Missing self-induced activation of NK cells
PBMCs were cultured overnight in RPMI supplemented with 500 UI/ml of recombinant human IL-2 (R&Dsystems). Purified NK cells (105 cells) were then mixed with endothelial cells at a ratio of 1:1 in flat-bottomed 96-well plates, centrifuged at 100 g for 1 minutes, and incubated at 37°C at 5% CO2. Anti-CD107a-FITC (clone H4A3, 5 µl, ThermoFisher Scientific) was added prior the start of the assay. One hour after the beginning of the co-culture, Golgi Stop (BD biosciences) was added to each well.
After 4 hours of co-culture, the cells were harvested and surface stained with appropriate antibody combinations to identify KIR subsets. The cells were subsequently fixed and permeabilized (Cytofix/Cytoperm fixation/permeabilization kit, BD Biosciences), stained with anti-MIP-1ß-V450 (clone D21-1351, 1/40, BD biosciences) antibodies and analysed by flow cytometry.
IL15-induced mTORC1 activation in NK cells
PBMCs of 24 patients diagnosed with a breast cancer were collected before and one month after the introduction of a mTOR inhibitor (everolimus). PBMCs were cultured for 1 hour in complete RPMI. When indicated, 100 ng/ml of IL-15 was added to the cultures. After 1 hour, the cells were harvested and surface stained with appropriate antibody combinations: anti-CD7 and anti-CD3. The cells were subsequently fixed and permeabilized (Cytofix/Cytoperm fixation/permeabilization kit, BD Biosciences), stained with anti-Phospho-S6 Ribosomal Protein Ser 235/236 (clone D57.2.2E, 1/50, Cell Signaling Technology, Leiden, The Netherlands) antibody and analysed by flow cytometry.

Endothelial flow cross match
To detect non-HLA anti-endothelial cell antibodies, we used a flow cross match technique. Briefly, target endothelial cells were HLA-matched to avoid false positive tests due to HLA binding (for sera containing anti-HLA antibodies that were not specific of kidney donor). Confluent endothelial cell monolayers were starved overnight in endothelial cell basal medium supplemented with 2% FBS without growth factors and incubated with recombinant human TNF α (100 U/mL, Peprotech) for 48 hours. Endothelial cells were then dissociated with trypsin and 1 to 2.105 endothelial cells were incubated for 30 minutes at room temperature with 25 µl of serum diluted at ¼ in PBS 1x FBS 1%. Reactivity of patient's sera for endothelial cells was revealed by incubation with a FITC-conjugated F(ab')2 anti-human IgG (clone 30242, Bio rad, Hercule, CA, USA) for 20 minutes at 4 °C. The fluorescence level was expressed as Mean Fluorescence Intensity. A serum containing an anti-HLA class I antibody directed against HLA typing of the endothelial cell lines was used as positive control. Negative controls were performed using a pool of human AB sera from healthy male donors.

Instrument

Sample acquisitions were made on a LSR FORTESSA or a FACScanto II® flow cytometer (BD biosciences)

Software

Analyses were performed with FlowJo software version 10.0.8r1 (Tree Star Inc, Ashland, OR, USA).

Cell population abundance

No sorting by flow cytometry was done for this study

Gating strategy

Gating strategy for analysis of HLA class I expression on endothelial cells and identification of non-HLA antibodies in patients' sera (endothelial flow cross matchs)
Morphologic gate on endothelial cells (SSC-A vs FSC-A)
Gate on single cells (FSC-H vs FSC-A)
Gate on viable cells (fixable vialibity dye vs FSC-A)

Gating strategy for NK cell and T cell count
Morphologic gate on lymphocytes (SSC-A vs FSC-A)
Gate on CD45+ cells (CD45 vs FSC-A)
Gate on NK cells and T cells(CD3 vs CD56 mAbs)

Gating strategy for human KIR phenotyping and Missing self-induced activation of NK cells
Morphologic gate on lymphocytes (SSC-A vs FSC-A)
Gate on single cells (FSC-H vs FSC-A)
Gate on viable cells (fixable vialibity dye vs FSC-A)
Gate on NK cells (CD3,CD14,CD19 versus CD56 mAbs)
Gate on KIR2DL1+ NK cells (KIR2DL1 vs KIR2DL1-S1 mAbs)
Gate on KIR2DL2+ and KIR2DL3+ NK cells (KIR2DL3 vs KIR2DL2-3-S2 mAbs)
Gate on KIR3DL1+ and KIR3DL2+ NK cells (KIR3DL1 vs KIR3DL1-2 mAbs)

Flow Cytometry

Plots

Confirm that:

☒ The axis labels state the marker and fluorochrome used (e.g. CD4-FITC).

☒ The axis scales are clearly visible. Include numbers along axes only for bottom left plot of group (a 'group' is an analysis of identical markers).

☒ All plots are contour plots with outliers or pseudocolor plots.

☒ A numerical value for number of cells or percentage (with statistics) is provided.

Gating strategy for analysis of p-S6RP expression in the blood of patients
Morphologic gate on lymphocytes (SSC-A vs FSC-A)
Gate on NK cells (CD7 vs CD3)

☒ Tick this box to confirm that a figure exemplifying the gating strategy is provided in the Supplementary Information.
